# Interaction of Soil Heavy Metal Pollution with Industrialisation and the Landscape Pattern in Taiyuan City, China

**DOI:** 10.1371/journal.pone.0105798

**Published:** 2014-09-24

**Authors:** Yong Liu, Chao Su, Hong Zhang, Xiaoting Li, Jingfei Pei

**Affiliations:** 1 Institute of Loess Plateau, Shanxi University, Taiyuan, Shanxi Province, China; 2 School of Mathematical Sciences, Shanxi University, Taiyuan, Shanxi Province, China; National Institute of Environmental and Health Sciences, United States of America

## Abstract

Many studies indicated that industrialization and urbanization caused serious soil heavy metal pollution from industrialized age. However, fewer previous studies have conducted a combined analysis of the landscape pattern, urbanization, industrialization, and heavy metal pollution. This paper was aimed at exploring the relationships of heavy metals in the soil (Pb, Cu, Ni, As, Cd, Cr, Hg, and Zn) with landscape pattern, industrialisation, urbanisation in Taiyuan city using multivariate analysis. The multivariate analysis included correlation analysis, analysis of variance (ANOVA), independent-sample T test, and principal component analysis (PCA). Geographic information system (GIS) was also applied to determine the spatial distribution of the heavy metals. The spatial distribution maps showed that the heavy metal pollution of the soil was more serious in the centre of the study area. The results of the multivariate analysis indicated that the correlations among heavy metals were significant, and industrialisation could significantly affect the concentrations of some heavy metals. Landscape diversity showed a significant negative correlation with the heavy metal concentrations. The PCA showed that a two-factor model for heavy metal pollution, industrialisation, and the landscape pattern could effectively demonstrate the relationships between these variables. The model explained 86.71% of the total variance of the data. Moreover, the first factor was mainly loaded with the comprehensive pollution index (*P*), and the second factor was primarily loaded with landscape diversity and dominance (*H* and *D*). An ordination of 80 samples could show the pollution pattern of all the samples. The results revealed that local industrialisation caused heavy metal pollution of the soil, but such pollution could respond negatively to the landscape pattern. The results of the study could provide a basis for agricultural, suburban, and urban planning.

## Introduction

From industrialized age, the unreasonable utilization of natural resources by humans caused many ecological and environmental issues, including land resources. Particularly, in the recent two decades, with the process of urbanization and industrialization, human activities, such as excessive use of fertilizers and pesticides, sewage irrigation, and discharge of waste influenced soil environment significantly. Soil in many regions has been polluted by various pollutants in different levels. Soil pollution could be harmful for human health. For example, it could cause toxicity, cancer, and gene mutation. Specifically, the soil heavy metal pollution is one of the most important issues because of the innate traits of heavy metals. Soil pollution by heavy metals may cause by waste water irrigation. The waste water is mainly from heavy industries discharging in urban areas. Pollution, products, and information are dispersed from urban area to suburban area. This process causes the pollution of the soil by heavy metals.

Landscape pattern is also the result of human activities. Landscape pattern is the mixture of natural and human-managed patches that vary in shape, size and arrangement. In other words, landscape pattern is the arrangement of landscape components with different sizes and shapes [1], [2]. Composition and configuration of landscape components are basic properties of landscape pattern [1], [2]. Composition describes the number and relative frequency of components, and configuration refers to the spatial arrangement of the landscape components [1], [2]. Landscape patterns result from complex interactions among social, biological, and physical forces [3]–[6]. Non-natural landscapes, e.g., agricultural landscapes, reflect not only natural foundations but also social conditions and human activities [7], [8]. Human activities strongly affect landscape, creating a mosaic of natural and human-managed patches [9]. Moreover, urban, suburban, and agricultural areas may interact with each other [6]. Suburban areas and agricultural landscapes are strongly influenced by the understanding and management of landscape patterns and structures [6].

Landscape indices, including patch shape, patch size, landscape diversity (*H*), relative richness (*R*), landscape aggregation (*C*), and dominance (*D*), have been widely used to describe landscape patterns. For example, landscape indices were used to analyse the spatial pattern of agricultural landscapes on the Loess Plateau [8]. In particular, the patch size index could represent the landscape pattern in a direct and clear way. Increases in the landscape diversity index reflect increases in the diversity of the landscape. The relative richness index, describing the richness of a landscape and dominance, can measure the extent to which one or a few patch types dominate a certain landscape [8], [10]. However, few previous studies have conducted a combined analysis of the landscape pattern and heavy metal pollution. The comprehensive consideration of landscape patterns and pollution may offer a new perspective for research in soil pollution. It expected to provide important basis for agricultural, suburban, and urban planning.

Multivariate analysis provides tools for classifying relationships among measured variables. Principal component analysis (PCA) and factor analysis are the two most common multivariate analysis methods. Principal component analysis, a statistical method, linearly transforms an original set of variables into a substantially smaller set of uncorrelated new variables that represent most of the information in the original data set [11], [12]. A smaller set of uncorrelated variables is easier to understand and apply in further analysis [11], [12]. Factor analysis is based on the assumption that certain underlying factors influence the covariation of the observed variables. Numbers of underlying factors are fewer than observed variables [11], [13]. Many ecological and environmental studies used PCA. For example, PCA was used to study soil pollution, was applied to surface water quality assessment, and was combined with the geoaccumulation index to assess heavy metal pollution of the soil in the vicinity of a copper mine tailings area, and so on [12], [14]–[17].

During the past two decades, urbanisation and industrialisation in Shanxi, especially in Taiyuan city, have polluted many agricultural areas by discharging waste water through irrigation systems. In this polluted area, the heavy metal pollution of the soil is one of the greatest concerns. This study used multivariate analysis to reveal the relationships of soil heavy metal pollution, industrialization, urbanization, and landscape pattern. Firstly, correlation analysis was used to explore the correlations among heavy metals. Secondly, ANOVA and independent-sample T test were applied to reveal the impact of industrialization on soil heavy metal pollution. Then, correlations between soil heavy metals and urbanization, landscape indices were analysed to describe the characteristics of heavy metal pollution of the soil and the patterns of urbanisation at the sampling sites. Finally, PCA was used to group the heavy metal pollution and urbanization, industrialization, landscape indices. These analyses aimed to identify the interactions among soil heavy metals, landscape pattern, and human activities.

The objectives of this paper are to: (1) illustrate the spatial distribution of the eight heavy metals in the soil by constructing maps of the suburban area of Taiyuan city; (2) explore the relationships between heavy metal pollution and landscape pattern, industrialisation; and (3) characterise the factor patterns of heavy metal pollution, landscape pattern, and industrial plants. This study was expected to provide a basis for preventing soil pollution and protecting the ecological environment in local area.

## Materials and Methods

### Ethics statement

The work does not require any permits or approvals of any authorities and does not involve endangered or protected species, the samples were collected from ordinary farmland, and those lands are not privately or protected.

### Sampling description

**Figure 1:**
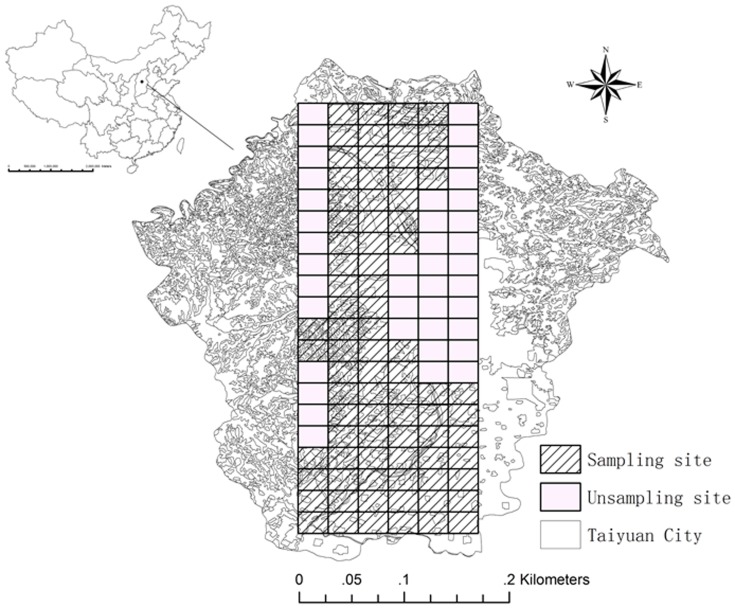
Study area and sampling sites.

The study was conducted in the suburban area of Taiyuan city, Shanxi, China (Fig. 1), except the west and east of Taiyuan. The west, east, and parts of north of Taiyuan city are highly mountainous, so that these areas couldn't be sampled. Besides, Fen River is the main source of drinking water and irrigation system of Taiyuan city, and it runs through the north and south in Taiyuan. Thus, the spatial domain is irregularly shaped, being much narrower in the East-West direction and much longer in the North-South direction. The longitude of the study area is from 112.57° E to 112.74° E and the latitude is from 36.62° N to 37.03° N. Taiyuan city is the capital of Shanxi province and is a base of heavy industry in China. Certain local sites may be polluted by waste water discharging from industrial plants through irrigation systems.

Samples were collected in 2008 from geographically distributed sites arranged in a network pattern. Eighty sampling sites were sampled in the research area (Fig. 1). All samples were taken between 6^th^ April, 2008 and 8^th^ April, 2008. The topsoil was sampled at depths of 0–20 cm. The average pH value of the topsoil was 8.6.

### Comprehensive pollution index

Table 1 lists the heavy metals that might be discharged with waste water from different types of plants [6]. Table 2 shows the background values for all heavy metals found in soils of Shanxi province [18].

**Table 1:** Industrial plants and heavy metals.

Metals	Description of sources
Pb	Batteries, glass, gasoline, cement, ammunition, paints, insecticides, pigments,
Cu	Brass, wire, alloys, dyes, plating, pipes, paints
Ni	Steel and alloys, cosmetics, electroplating, batteries, pigments
As	Pesticides, glass, pigments, wood preservatives, fireworks, textiles, printing, lubricating oil, alloys, oil cloth, semiconductors, photo-conductors
Cd	Electroplating, alloys, cement, pigments, batteries, plastics, rubber, textiles
Cr	Pigments, electroplating, chrome-plating, chrome, varnishes, dye fixers, tanning, photography emulsion
Hg	Paints, fungicides, plastics, paper products, cement, catalysts, pharmaceuticals, batteries
Zn	Alloys, paints, glass, textiles, rubber, metal coatings, cosmetics

**Table 2:** Background content of heavy metals in soils in Shanxi province (mg/kg).

Metals	Pb	Cu	Ni	As	Cd	Cr	Hg	Zn
Background content	14.7	22.9	29.9	9.1	0.102	55.3	0.023	63.5

The Comprehensive Index Evaluation method was applied to measure the comprehensive pollution index (*P*) of each sampling site.

*C_i_* is the measured value for heavy metal *i* and *C_i0_* is the background value of Shanxi province for heavy metal *i* in the soils [18]; *i* ranges from 1 to 8. Then, 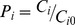
(1)


Comprehensive pollution index *P* is given by [19]

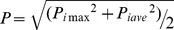
(2)where *P_i_*_max_ is the maximum value of *P_i_* in all sites and *P_iave_* is the average value of *P_i_* in all sites.

### Landscape indices

**Figure 2:**
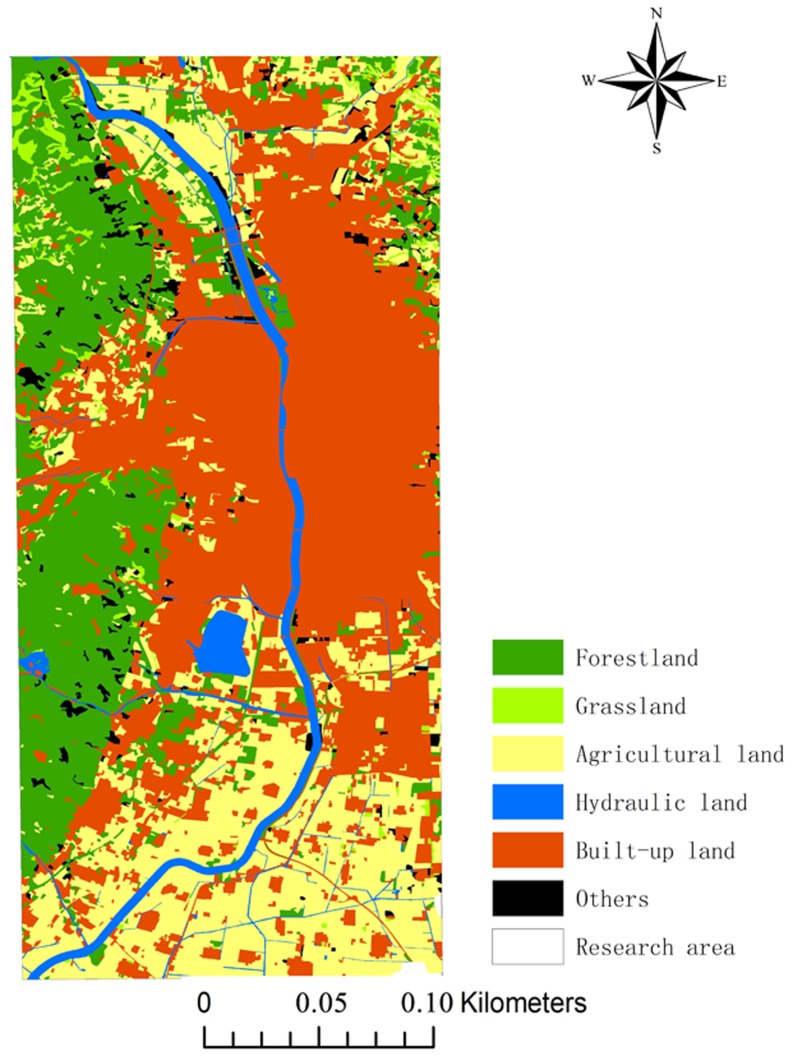
Land use in the study area.

The following landscape indices were used to describe the landscape pattern for each sampling site: landscape diversity (*H*), relative richness (*R*), dominance (*D*). Fig. 2 shows the land use types of Taiyuan city in 2008. Six land use types–green space, hydraulic, built-up, industrial, agricultural, and others–were used to calculate the landscape indices for 80 sampling sites by ArcGIS 10.0 [20].

The landscape diversity index used was the Shannon-Weaver Diversity (*H*) [8], 
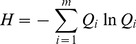
(3)where *Q_i_* is the proportion of land use type *i* at a site and *m* is the number of observed land use types.

Relative richness (*R*) is given by [21]

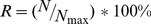
(4)where *N* is the number of different land use types present and *N*_max_ is the maximum number of land use types possible. The larger the value of *R* is, the richer the landscape will be.

Dominance (*D*) could measure the extent to which one or a few patch types dominate the landscape, and it is defined as [8]

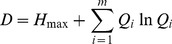
(5)where *H_max_* is the maximum value of diversity among all the sites, *m* is the number of landscape types and *Q_i_* is the proportion of area of type *i*.

### Industrialisation indices

The following industrialisation indices were used to describe the landscape pattern for each sampling site (Table 3).

**Table 3:** The industrialisation indices.

The industrialisation index	Representation
*NEP*	the number of electroplating plants
*NP1*	the number of power plants
*NCM*	the number of coal and mineral plants
*NP2*	the number of paper plants
*NC*	the number of chemical industries
*NM*	the number of metal plants
*NP*	the total number of industrial plants
*Rup*	the urban planning area ratio

*Rup* is the proportion of each sampling site covered by urban planning area, it is calculated from:
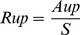
(6)where *Aup* is the urban planning area at the sampling site and *S* is the area of the sampling site.

The industrialisation indices for 80 sampling sites were calculated using ArcGIS 10.0 [20].

**Figure 3:**
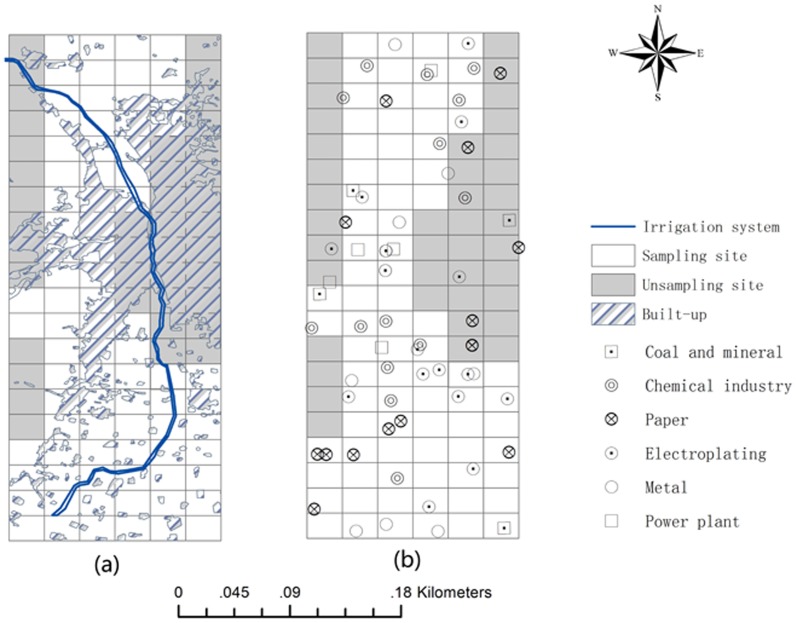
Spatial distribution of: (a) urban planning area and (b) industrial plants.

Fig. 3(a) shows the urban planning area in research area, corresponding to the built-up zone. The highly urbanised and industrialised area located in the centre of Taiyuan. The Fen River flows through Taiyuan. Fig. 3(b) shows the location of the major large-scale industries in the study area. The pollutant emissions from these industries were more than 80% of the total emissions in the study area. Industrial plants were evenly distributed among the sampling sites. Several paper plants, two chemical plants, and one electroplating plant were located at sites that were not sampled (Fig. 3 (b)). It is possible that these industrial plants could have affected the concentrations of heavy metals at the sampling sites.

### Statistical analysis

Descriptive statistics were used to identify the main features of the concentrations of heavy metals. Descriptive statistics are used to summarise the general trends in a data set. This analysis is fundamental in statistical studies. The indicators used in this study included mean, standard deviation, range for each heavy metal, CV% value, and correlation analysis.

Pearson correlation analysis was used to measure the correlations between two numeric variables. Analysis of variance (ANOVA) and independent-sample T test were applied to test if the industrialization had significant influences on soil heavy metal pollution. They were performed with SPSS 18.0 statistical software.

The principal component analysis was performed with **SIMCA-P software**. In this method, the first factor explains the greatest variation in the variables of interest, the second factor explains the next highest amount of variation and the other factors similarly explain the remaining variation [15], [16], [22]. Finally, the factor scores are calculated from the variables using a regression method with a matrix of factor-score coefficients [15].

In this study, PCA was applied to explore the factor pattern of soil heavy metal (Pb, Cu, Ni, As, Cd, Cr, Hg, and Zn) pollution at the 80 sampling sites. The comprehensive pollution index, industrialization indices, and landscape indices were then grouped using PCA to show the relationships among them.

## Results

### Descriptive statistical analysis of eight heavy metals

The mean, standard deviation, and range for each heavy metal are presented in Table 4. The CV% values reflect the mean variation of each sampling site in the population. The order of the CV%s for each element, from high to low, was Hg>Pb>As>Cd>Cu>Ni>Zn>Cr. This result showed that the variation of Hg and Pb in the soil was larger than other metals.

**Table 4:** Statistics of the concentrations of eight heavy metals (mg/kg).

Metal	Pb	Cu	Ni	As	Cd	Cr	Hg	Zn
*N*	80	80	80	80	80	80	80	80
Mean	26.24	28.87	29.76	10.96	0.21	73.69	0.12	86.08
Standard deviation	10.70	5.28	5.10	2.81	0.05	9.22	0.08	11.68
Median	23.95	27.90	29.90	11.15	0.20	72.55	0.10	83.89
Maximum	86.00	47.60	50.2	17.80	0.40	109.00	0.39	121.00
Minimum	17.40	17.20	20.5	4.50	0.10	54.70	0.02	58.60
CV (%)	0.409	0.184	0.172	0.258	0.248	0.126	0.628	0.137

**Figure 4:**
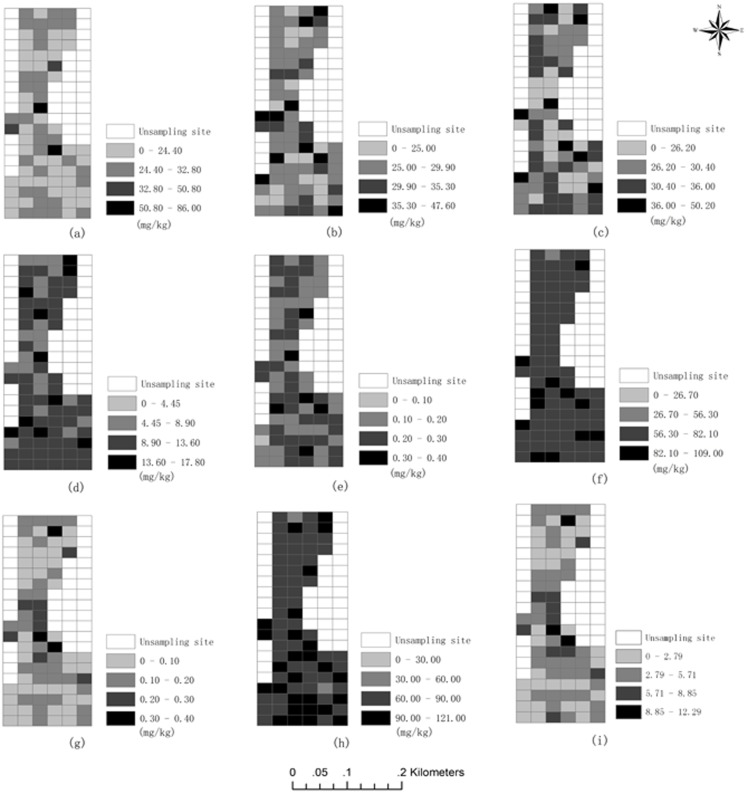
Spatial distributions of heavy metals in soil and comprehensive pollution index: (a) Pb, (b) Cu, (c) Ni, (d) As, (e) Cd, (f) Cr, (g) Hg, (h) Zn and (i) *P*.

Fig. 4 shows the spatial distribution of the measured values for the eight heavy metals at each sampling site and the spatial distribution of the comprehensive pollution index (*P*). It shows that the concentrations of Pb, Cu, and Hg in the centre of the study area were higher than in other areas. The concentrations of Cr and Zn were uniformly distributed, and the concentrations of Ni, As, and Cd were randomly distributed throughout the domain.

Fig. 4 (i) shows the spatial distribution of the comprehensive pollution index values for the sampling sites. This pattern was similar to the spatial distribution pattern of the heavy metal Hg. The most severely polluted sites were those of highest Hg concentration. These results showed that the concentrations of Hg substantially exceeded the background value, and Hg pollution is relatively serious.

### Relationships between landscape indices and industrialisation

**Figure 5:**
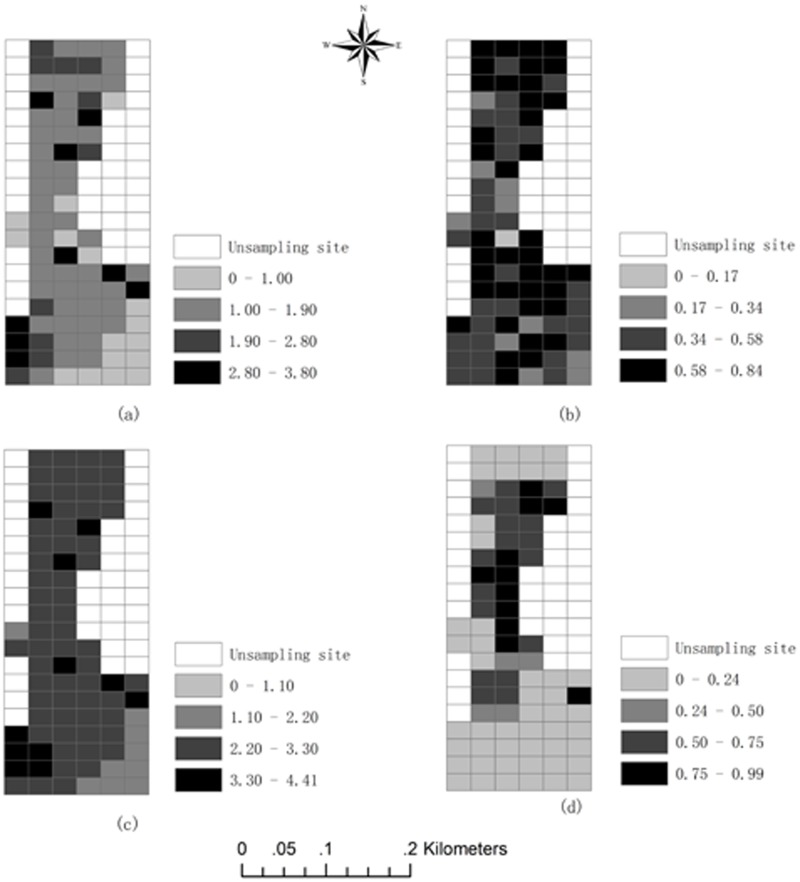
Spatial distribution of: (a) landscape diversity, (b) relative richness, (c) landscape dominance, and (d) urban planning ratio.

The spatial distribution of landscape diversity, relative richness, landscape dominance and the urban planning ratio at the sampling sites were shown in Fig. 5. The sampling sites with greater landscape diversity and lower landscape dominance located in the central and south-western parts of the study area (Fig. 5 (a) and (c)). Sampling sites with greater landscape dominance were primarily in the south-eastern portions of the study area (Fig. 5 (c)). The sites with high values of relative richness were located in the northern and central parts of the study area (Fig. 5 (b)). The sites with high values of the urban planning ratio were located in the centre of the study area (Fig. 5 (d)). The comparison of the relative richness, landscape diversity, and landscape dominance maps (Fig. 5 (a)–(c)) illustrates that the landscape diversity showed a more significant pattern than the relative richness and dominance. The spatial distribution of the urban planning ratio appears to display a north-south orientation corresponding to the urban planning area (Fig. 3 (a)). Higher values of the urban planning ratio were found in the central sampling sites. These sites are located near the centre of Taiyuan.

**Figure 6:**
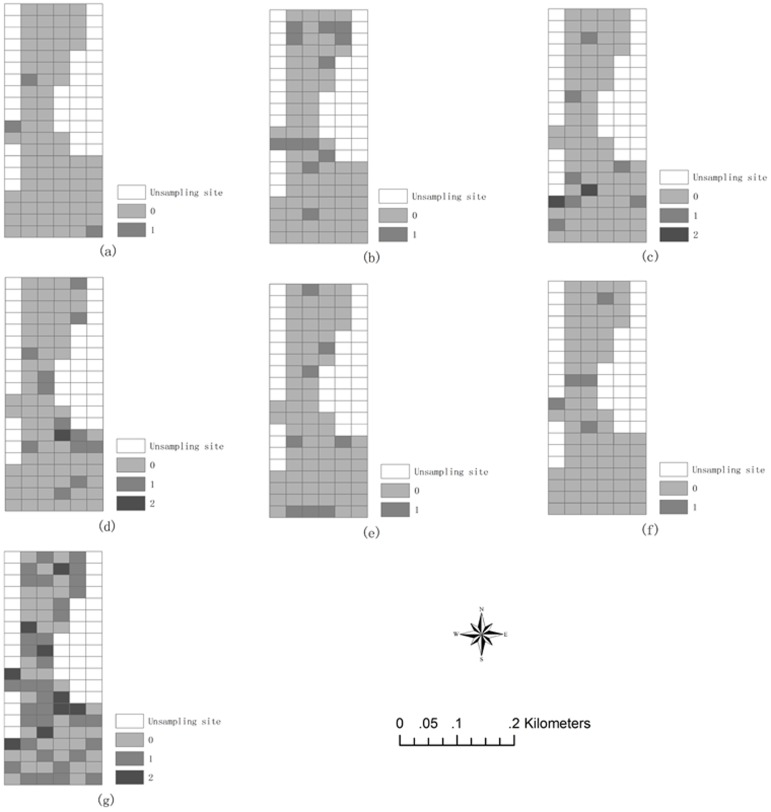
Number of: (a) coal and mineral industrial plants, (b) chemical industrial plants, (c) paper industrial plants, (d) electroplating industrial plants, (e) metal industrial plants and (f) power plants, and (g) total number of industrial plants at each sampling site.

Fig. 6 displays the number of each type of pollution source by sampling site. These values range from 0 to 2 because the study area was part of the urban district and was the site of relatively few large industries, which usually located primarily in suburban districts close to the study area. This distribution pattern was similar to the spatial distribution pattern of the eight heavy metals (Fig. 4).

### Correlations among the eight heavy metals

The Pearson Correlation was conducted in SPSS 18.0 [23]. The Pearson correlations among the eight heavy metals were shown in Table 5. The correlations among the eight heavy metals were generally significant. The correlations among Pb, Cu, Ni, As, Cr, and Zn were highly significant (*Sig*.<0.01), whereas the correlations between Cd and the other heavy metals were not significant. Additionally, Hg was positively correlated with Pb and Cu (*Sig*.<0.01), and negatively correlated with Ni and Cr (*Sig*.<0.05).

**Table 5:** Correlations among the eight heavy metals.

	Pb	Cu	Ni	As	Cd	Cr	Hg	Zn
Pb	1.00							
Cu	0.598**	1.00						
Ni	0.269**	0.425**	1.00					
As	0.290**	0.493**	0.509**	1.00				
Cd	0.225*	0.174	0.078	0.075	1.00			
Cr	0.101	0.377**	0.337**	0.477**	0.071	1.00		
Hg	0.392**	0.300**	−0.234*	−0.204	0.022	−0.228*	1.00	
Zn	0.671**	0.735**	0.308**	0.391**	0.245*	0.370**	0.349**	1.00

** *Sig.*<0.01 (two-tailed),

* *Sig.*<0.05 (two-tailed).

### Effects of industrialization on soil heavy metal pollution

The variables, the number of industrial plants, were ordinal variables. We used analysis of variance (ANOVA) and independent-sample T test to analyze if the industrial plants had significant effects on soil heavy metal pollution [23]. The results were shown in Table 6.

**Table 6:** Grouping mean concentrations of heavy metals and significance tests of differences among industrialisation indices.

Grouping variable			Pb	Cu	Ni	As	Cd	Cr	Hg	Zn	*P*
*NP*	0		22.85a	27.11b	29.24	10.77	0.19a	71.86	0.10b	81.82a	3.18b
	1		28.04a	29.48a	30.01	11.11	0.23a	74.88	0.13a	88.88a	4.37a
	2		33.37a	33.84a	30.92	11.14	0.22a	76.74	0.18a	93.00a	5.91a
*Sig.*(ANOVA)			0.012*	0.001**	0.634	0.860	0.017*	0.228	0.004**	0.006**	0.003**
*NEP*	0		24.66	28.13	29.35	10.69	0.20	73.13	0.11	84.20	3.79
	1		30.07	31.50	31.26	11.87	0.24	75.80	0.15	93.91	4.96
*Sig.* (T-test)			0.317	0.028*	0.232	0.173	0.116	0.359	0.277	0.005**	0.258
*NP1*	0		26.31	28.91	29.81	10.99	0.21	73.99	0.12	85.74	4.05
	1		25.94	27.46	27.87	9.70	0.23	69.90	0.11	87.36	3.70
*Sig.* (T-test)			0.933	0.495	0.341	0.244	0.190	0.271	0.729	0.733	0.718
*NCM*		0	26.09	28.47	29.56	10.93	0.21	73.12	0.12	85.78	3.98
		1	30.00	39.17	34.73	11.67	0.22	88.47	0.14	93.80	4.48
*Sig.* (T-test)			0.540	0.00**	0.087	0.661	0.727	0.279	0.734	0.249	0.725
*NP2*	0		26.11	28.74	29.94	11.15	0.21	73.11	0.12	86.01	3.82
	1		28.06	30.94	27.04	8.08	0.20	82.46	0.21	87.10	6.63
*Sig.* (T-test)			0.698	0.372	0.224	0.018*	0.706	0.395	0.010*	0.843	0.010*
*NC*	0		26.03	28.59	29.99	10.96	0.21	74.04	0.11	85.89	3.68
	1		27.43	30.48	28.44	10.95	0.21	71.73	0.18	87.18	5.78
*Sig.* (T-test)			0.681	0.257	0.339	0.992	0.819	0.431	0.112	0.735	0.111
*NM*	0		26.23	29.02	29.57	11.03	0.21	73.63	0.12	85.93	4.00
	1		26.31	27.56	31.40	10.35	0.22	74.21	0.12	87.46	3.89
*Sig.* (T-test)			0.983	0.465	0.343	0.526	0.555	0.912	0.903	0.728	0.891

** *Sig.*<0.01 (two-tailed),

* *Sig.*<0.05 (two-tailed).

Letters (a, b) indicated the results of Bonferroni test (*Sig.*<0.01), no significant differences (same letter) or significant differences (different letter).

The results of one-way ANOVA showed that the variable *NP* had highly significant effects on Cu, Hg, Zn, and *P* (*Sig.*<0.01), and significant effects on Pb, Cd (*Sig.*<0.05). Furthermore, Bonferroni multiple comparisons test was performed at 0.01 level after the ANOVA (Table 6). It revealed that the grouping means of Cu, Hg and the comprehensive value *P* in the sites with more industrial plants were significantly higher than those with less industrial plants.

For variables *NEP*, *NP1*, *NCM*, *NP2*, *NC* and *NM*, independent-sample T test was used to explore the relationships between them and soil heavy metal pollution. Table 6 indicated that, the variable *NEP* had significant effects on heavy metals Cu (*Sig.*<0.05) and Zn (*Sig.*<0.01). The variable *NCM* had a significant influence on heavy metal Cu (*Sig.*<0.01). And the variable *NP2* had significant influences on heavy metals As, Hg, and comprehensive pollution index *P* (*Sig.*<0.05). In other words, the number of electroplating plants had significant influences on concentrations of Cu and Zn. The number of coal and mineral plants had a significant influence on concentration of Cu, and the number of paper plants had significant influence on concentrations of As, Hg, and value of *P*. However, the variables *NP1*, *NC*, and *NM* had no significant effects on any heavy metal (*Sig.*>0.05).

In conclusion, the total number of industrial plants had highly significant effects on many heavy metals (Cu, Hg, and Zn) and the comprehensive pollution index *P*. Cu was mainly affected significantly by the number of electroplating plants and coal and mineral plants. And the number of electroplating plants also had a significant influence on concentration of Zn. The number of paper plants had significant influence on concentrations of As, Hg, and value of *P*.

### Relationships of heavy metals and landscape pattern, urbanization

The Pearson correlation coefficients of the heavy metals with the landscape indices were shown in Table 7. These coefficients were used to identify the relationships between the pairs of variables analysed for the 80 sampling sites.

**Table 7:** Correlations of the heavy metals with landscape indices.

	Pb	Cu	Ni	As	Cd	Cr	Hg	Zn	*P*
*H*	−0.178	−0.180	−0.077	−0.073	−0.200	−0.100	−0.294**	−0.330**	−0.296**
*R*	−0.172	−0.110	0.100	−0.011	−0.l25	0.003	−0.141	−0.141	−0.108
*D*	0.178	0.180	0.077	0.073	0.200	0.100	0.294**	0.330**	0.296**
*Rup*	0.015	−0.093	−0.226*	−0.048	−0.029	−0.195	0.226**	−0.105	0.223*

** *Sig.*<0.01 (two-tailed),

* *Sig.*<0.05 (two-tailed).

Table 7 shows that the variables *H*, *D*, and *Rup* had significant correlations with Hg, Zn, and *P*. According to the two-tailed test, the correlation coefficients of Hg with *H*, *D*, and *Rup* were −0.294, 0.294, and 0.226, respectively (*Sig.*<0.01). It reflected a significant relationship. The landscape diversity had a significant negative correlation with Hg, and the urban planning ratio had a significant positive correlation with Hg. Similarly, Zn showed significant correlations with the landscape indices. The correlation coefficients of Zn with *H* and *D* were −0.330 and 0.330, respectively (*Sig.*<0.01). Zn also had a significant negative correlation with landscape diversity. Besides, the comprehensive pollution index *P* had a significant correlation with *H*, *D*, (*Sig.*<0.01), and *Rup* (*Sig.*<0.05). As with Hg, the variable *P* also had a negative correlation with landscape diversity and a positive correlation with urban planning ratio. The possible reason for the correlations between the heavy metals Zn, Hg and the indices *H*, *D* was that fewer industrial plants were located in areas with higher diversity and lower dominance.

In conclusion, the heavy metal Hg showed significant negative correlations with landscape diversity, as did Zn. These results showed that increases in landscape diversity could potentially mitigate the soil pollution caused by heavy metals. Hg was significantly correlated with the urban planning ratio. This result showed that urbanisation may be related to higher concentrations of Hg.

### Relationships among landscape diversity, urbanization and industrialization

**Figure 7:**
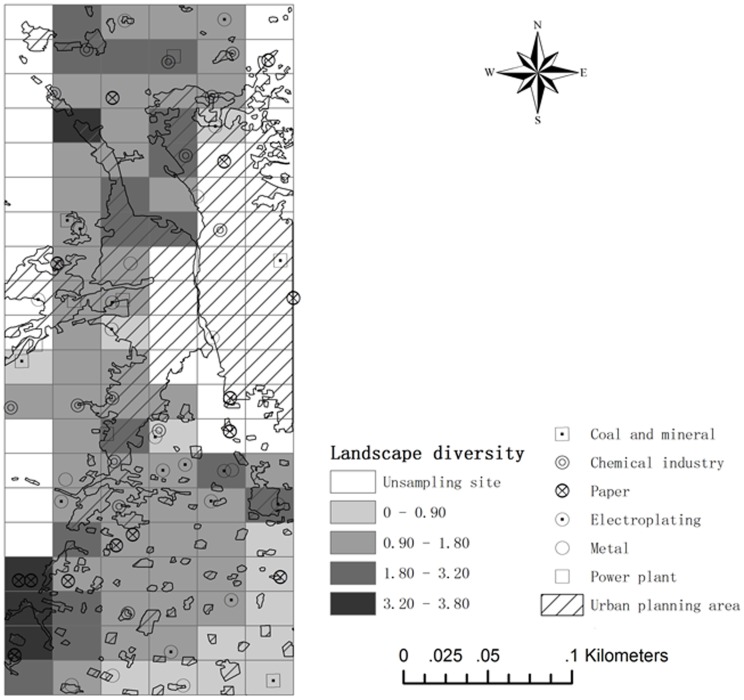
Overlay map of landscape diversity, industrial plants and the urban planning area.

Fig. 7 shows the overlay map combining landscape diversity (*H*), the urban planning area, and industrial plants to explore their relationships. Because the variables *H* and *Rup* are numeric variables, the Pearson correlation analysis can be done between them. But the correlation between them was not significant (*Sig.*>0.05) and the plot between them didn't have an obvious trend. However, in Fig. 7, it divided the landscape diversity index into four classes, and it could be seen that the four sites with the fourth landscape diversity class didn't have much higher urban planning ratio. In order to analyze their relationship further, we analyzed if the division has significant influences on *Rup* using ANOVA (Analysis of variance) [23]. The results showed there was no significant influence (*Sig.*>0.05), but the group mean values showed that the second and third classes had the much higher urban planning ratio. In addition, this map also shows that most industrial plants were located in non-urbanised areas. Several plants were located on the boundaries of the urban planning area. These results showed that industrialisation was not always highly corresponding to urbanisation. It reflected the policy of sustainable development.

### PCA of soil heavy metal pollution, landscape and industrialisation

A Principal Component Analysis (PCA) provides an overview of the lower-dimensional structure of multivariate data and can reduce the dimensionality of a complex data set to facilitate more effective analysis. In this paper, its process was conducted in SIMCA-P [24]. In this process, the raw data had been centralized (File S1). The centralization method is subtracting mean value from original data. The aim is to integrate different variables data scale.

**Figure 8:**
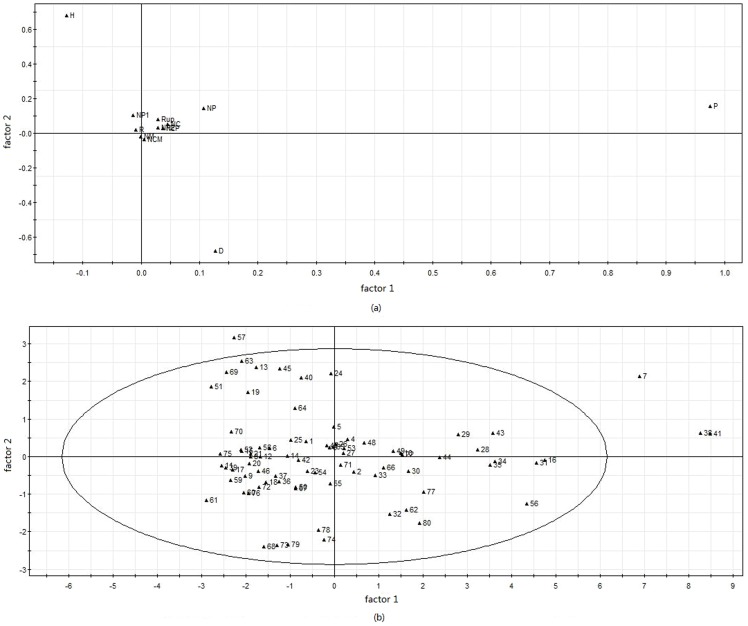
PCA for soil heavy metal pollution, industrialisation and landscape pattern.

PCA showed that a two-group model explained 86.71% of the total variance of all the variables (*P*, *H*, *R*, *D*, *Rup*, *NEP*, *NP1*, *NCM*, *NP2*, *NC*, *NM*, and *NP*). This result is highly satisfactory. The first component explained 71.23% of the total variance. Fig. 8 (a) shows a plot of the 12 variables against their values on principal axes *X* and *Y*. The first factor reflected the comprehensive pollution index (*P*), and the second factor primarily reflected landscape diversity and dominance (*H* and *D*). But the loading of landscape diversity is positive and that of the other is negative. Other variables plotted closer to the original point. This showed that the contributions of these variables were quite small.

Fig. 8 (b) shows the PCA scores for the 80 sampling sites. A relatively large number of sampling sites were negative on the first factor. And a relatively large number of sampling sites were positive on the second factor. Because the origin represents the average values of all samples, these results showed that the comprehensive pollution index *P* of all the samples was higher than the mean value. Additionally, the landscape diversity was higher than the mean value and the dominance of most samples was lower than the mean value. The highest score on the first factor was that of sample 41. The second highest score on the first factor was sample 38, in the centre of the study research area. The sampling sites scoring highest on the second factor was 57, followed by 63, in the central to lower portion of the study area. These results were consistent with the relatively high values of the comprehensive pollution index for samples 41 and 38, and the relatively high landscape diversity and low dominance value of sample 57. Except for samples 41, 38, 7, and 57, other samples could be clustered to form a single class.

## Conclusions and Discussion

This paper used correlation analysis, principal component analysis, and geographic information system methodology to investigate and understand the impacts of landscape pattern, industrialisation, and urbanisation on the soil heavy metal pollution. Combining landscape and industrialisation indices and the urbanisation ratio, this study revealed the relationships. The landscape diversity, relative richness, and landscape dominance indices could effectively represent the landscape pattern of the study area. It is possible that other landscape indices, e.g., fractal dimension and landscape evenness, could also serve to describe this pattern. In this study, the data on the number of coal and mineral industries, chemical industries, paper plants, electroplating plants, metal industries, power plants, industrial plants, and the urban planning ratio played a important role in the spatial analysis of the pattern of industrialisation and urbanisation as well as in the multivariate analyses.

The analysis of the correlations among the heavy metals showed that the correlations among these variables were significant. Moreover, the correlations among Pb, Cu, Ni, As, Cr, and Zn were extremely significant. The exploration of the relationships between heavy metals and industrialisation, landscape indices showed that industrialisation could remarkably affect the concentrations of some heavy metals, such as Cu, Cd, Hg, Zn, and *P*. However, the landscape pattern (landscape diversity) was significantly negatively correlated with the concentrations of heavy metals, Hg, Zn, and *P* in the soil. This result is not consistent with the results of Lin et al., who investigated the relationship between heavy metals in the soil and the landscape pattern (landscape diversity, fractal dimension and landscape dominance) in Changhua County in Taiwan in 2002. A possible explanation of this discrepancy is that fewer plants were located at the sites with higher landscape diversity. Because there were fewer industrial plants in the area with a high urban planning ratio, the heavy metal pollution of the soil was not serious there. Additionally, the heavy metal pollution of the soil was affected by human activities in the study area.

Finally, the PCA showed that a two-factor model for heavy metals, industrialisation, and the landscape indices could effectively reveal the relationships of heavy metal pollution of the soil with industrialisation and the landscape pattern. The first factor was mainly loaded with the comprehensive pollution index (*P*), explaining 71.23% of the total variance, and the second factor was primarily loaded with landscape diversity and dominance (*H* and *D*). Other variables (*NEP*, *NP1*, *NCM*, *NP2*, *NC*, and *NM*) were closer to the original point. In future research, the number of industrial plants should be the subject of a more comprehensive investigation. The analysis of 80 sampling sites showed that (with the exception of samples 41, 38, 7, and 57) the samples could be clustered to form a single class. The comprehensive pollution index of samples 41 and 38 was relatively high, and the landscape diversity of sample 57 was the highest of all the samples and the dominance of 57 is the lowest.

## Supporting Information

File S1 **The raw data for the PCA.**
(DOC)

## References

[1] Han M, Sun YN, Xu SG, Tang XL (2005) Study on changes of marsh landscape pattern in Zhalong wetland assisted by RS and GIS. Progress in Geography 6: 42–49.

[2] Chen LD, Liu Y, Lu YH, Feng XM, Fu BJ (2008) Pattern analysis in landscape ecology: progress, challenges and outlook. Acta Ecologica Sinica 11: 5521–5531.

[3] Forman RTT, Godron M (1986) Landscape Ecology. Wiley: New York.

[4] Turner MG (1990) Landscape changes in nine rural counties in Georgia. Photogram. Eng. Remote Sens 3: 379–386.

[5] Hulshoff RM (1995) Landscape indices describing a Dutch landscape. Landscape Ecol 2: 101–111.

[6] Lin YP, Teng TP, Chang TK (2002) Multivariate analysis of soil heavy metal pollution and landscape pattern in Changhua county in Taiwan. Landscape and Urban Planning 2002: 19–35.

[7] Urban DL, O'Neill RV, Shugart HH (1987) Landscape ecology. Bioscience 37: 119–127.

[8] Fu BJ, Chen LD (2000) Agricultural landscape spatial pattern analysis in the semiarid hill area of the loess plateau, China. J. Arid Environ 3: 291–303.

[9] Leduc A, Prairie Y, Bergeron Y (1994) Fractal dimension estimates of a fragmented landscape: source of variability. Landscape Ecol 4: 279–286.

[10] O'Neill RV, Krummel JR, Gardner RH, Sugihara G, Jackson B, et al (1988) Indices of landscape pattern. Landscape Ecol 2: 153–162.

[11] Lewis-Beck MS (1994) Factor analysis and Related Techniques. SAGE Publications, Toppan Publishing, London

[12] Andrade JM, Kubista M, Carlosena A, Prada D (2007) 3-way characterization of soils by Procrustes rotation, matrix-augmented principle components analysis and parallel factor analysis. Analytica Chimica Acta 2007: 20–29. 10.1016/j.aca.2007.09.043 17950053

[13] Liu PWG (2009) Simulation of the daily average PM10 concentrations at Ta-Liao with Box-Jenkins time series models and multivariate analysis. Atmospheric Environment 2009: 2104–2113.

[14] Briz-Kishore BH, Murali G (1992) Factor analysis for revealing hydrochemical characteristics of a watershed. Environ Geol 19: 3–9.

[15] Carlon C, Critto A, Marcomini A, Nathanail P (2001) Risk based characterization of contaminated industrial site using multivariate and geostatistical tools. Environ Pollut 111: 417–427. 11202746 10.1016/s0269-7491(00)00089-0

[16] Roger LO, Rick WC, Jim CL (2012) Water quality sample collection, data treatment and results presentation for principle components analysis – literature review and Illinois River watershed case study. Water Research 2012: 3110–3122. 10.1016/j.watres.2012.03.028 22487543

[17] Wei ZY, Wang DF, Zhou HP, Qi ZP (2011) Assessment of Soil Heavy Metal Pollution with Principle Component Analysis and Geoaccumulation Index. Procedia Environment Sciences 2011: 1946–1952.

[18] Shi CW, Zhao LZ, Guo XB, Gao S, Yang JP, et al (1994) Background values of soil elements in Shanxi and there distribution feature. Jour Geol & Min Res North China 2: 188–196.

[19] Liu Y (2008) The research of land ecological risk assessment based on spatial information technology. Doctor's dissertation

[20] Song XD, Niu XY (2007) Geographic information system practice tutorial. Beijing: Science Press.

[21] Turner MG (1989) Landscape ecology: the effect of pattern on process. Annual Review on Ecological Systems 20: 171–197.

[22] Zhang H, Lu YL, Dawson RW, Shi YJ, Wang TY (2005) Classification and ordination of DDT and HCH in soil samples from the Guanting Reservoir, China. Chemosphere 2005: 762–769. 10.1016/j.chemosphere.2005.04.023 15936798

[23] Zhang WT, Kuang CW (2011) SPSS statistical analysis foundation. Beijing: Higher Education Press. pp. 265–342.

[24] Wang HW, Wu ZB, Meng J (2006) Linear and nonlinear approach of partial least square regression. Beijing: National Defence Industry Press.

