# Supplementary material for: Interaction of Soil Heavy Metal Pollution with Industrialisation and the Landscape Pattern in Taiyuan City, China
Source: PLoS One. 2014 Sep 24;9(9):e105798. doi: 10.1371/journal.pone.0105798 (PMC4174504; doi:10.1371/journal.pone.0105798)
Supplement: File S1 — The raw data for the PCA. (DOC) [file pone.0105798.s001.doc]

The raw data for the PCA

| ID | *NCM* | *NC* | *NP1* | *NEP* | *NM* | *NP2* | *NP* | *H* | *R* | *D* | *Rup* | *P* |
| --- | --- | --- | --- | --- | --- | --- | --- | --- | --- | --- | --- | --- |
| 1 | 0 | 0 | 0 | 0 | 0 | 0 | 0 | 1.9528 | 0.8333 | 1.8176 | 0.0326 | 3.5438 |
| 2 | 0 | 0 | 0 | 0 | 1 | 0 | 1 | 1.1734 | 0.8333 | 2.5970 | 0.0326 | 4.3485 |
| 3 | 0 | 0 | 0 | 0 | 0 | 0 | 0 | 1.7863 | 0.8333 | 1.9841 | 0.0023 | 4.0518 |
| 4 | 0 | 0 | 0 | 1 | 0 | 0 | 1 | 1.7863 | 0.8333 | 1.9841 | 0.0296 | 4.3147 |
| 5 | 0 | 1 | 0 | 0 | 0 | 0 | 1 | 2.0370 | 0.6667 | 1.7334 | 0.0303 | 4.0531 |
| 6 | 0 | 0 | 0 | 0 | 0 | 0 | 0 | 1.9252 | 0.5000 | 1.8452 | 0.0045 | 2.6891 |
| 7 | 0 | 1 | 0 | 0 | 0 | 1 | 2 | 2.0933 | 0.6667 | 1.6771 | 0.0071 | 11.0137 |
| 8 | 0 | 1 | 0 | 0 | 0 | 0 | 1 | 1.6906 | 0.6667 | 2.0798 | 0.0125 | 2.0427 |
| 9 | 0 | 1 | 0 | 0 | 0 | 0 | 1 | 1.3082 | 0.6667 | 2.4622 | 0.5000 | 1.7997 |
| 10 | 0 | 0 | 1 | 0 | 0 | 0 | 1 | 1.2833 | 0.6667 | 2.4871 | 0.6920 | 5.4501 |
| 11 | 0 | 0 | 0 | 0 | 0 | 0 | 0 | 1.6749 | 0.8333 | 2.0955 | 0.8586 | 1.5067 |
| 12 | 0 | 1 | 0 | 0 | 0 | 0 | 1 | 1.6335 | 0.5000 | 2.1369 | 0.6257 | 2.2270 |
| 13 | 0 | 0 | 0 | 0 | 0 | 0 | 0 | 3.4582 | 0.3333 | 0.3122 | 0.7463 | 2.7703 |
| 14 | 0 | 0 | 0 | 0 | 0 | 0 | 0 | 1.6967 | 0.5000 | 2.0737 | 0.6493 | 3.0227 |
| 15 | 0 | 0 | 0 | 0 | 0 | 0 | 0 | 1.9038 | 0.6667 | 1.8666 | 0.8604 | 2.0243 |
| 16 | 0 | 0 | 0 | 1 | 0 | 0 | 1 | 0.8372 | 0.8333 | 2.9332 | 0.8250 | 8.6200 |
| 17 | 0 | 0 | 0 | 0 | 0 | 0 | 0 | 1.6088 | 0.5000 | 2.1616 | 0.0877 | 1.7551 |
| 18 | 0 | 0 | 0 | 0 | 0 | 0 | 0 | 1.2547 | 0.5000 | 2.5157 | 0.6524 | 2.4166 |
| 19 | 0 | 1 | 0 | 0 | 0 | 0 | 1 | 2.8786 | 0.6667 | 0.8918 | 0.6770 | 2.2737 |
| 20 | 0 | 0 | 0 | 0 | 0 | 0 | 0 | 1.6918 | 0.6667 | 2.0786 | 0.0175 | 2.1671 |
| 21 | 0 | 0 | 0 | 0 | 0 | 0 | 0 | 1.8275 | 0.5000 | 1.9429 | 0.7353 | 2.2192 |
| 22 | 0 | 0 | 0 | 0 | 1 | 0 | 1 | 1.3516 | 0.5000 | 2.4188 | 0.6448 | 5.4851 |
| 23 | 1 | 0 | 0 | 1 | 0 | 0 | 2 | 1.1799 | 0.6667 | 2.5905 | 0.7217 | 3.1016 |
| 24 | 0 | 0 | 0 | 0 | 0 | 0 | 0 | 3.1381 | 0.5000 | 0.6323 | 0.8404 | 4.4095 |
| 25 | 0 | 0 | 0 | 0 | 0 | 0 | 0 | 1.9889 | 0.6667 | 1.7815 | 0.6183 | 3.1896 |
| 26 | 0 | 0 | 1 | 0 | 0 | 0 | 1 | 1.6211 | 0.3333 | 2.1493 | 0.9838 | 4.0241 |
| 27 | 0 | 0 | 0 | 0 | 1 | 0 | 1 | 1.5185 | 0.6667 | 2.2519 | 0.8286 | 4.1726 |
| 28 | 0 | 0 | 0 | 0 | 0 | 1 | 1 | 1.2061 | 0.5000 | 2.5643 | 0.7480 | 7.1694 |
| 29 | 0 | 0 | 0 | 1 | 0 | 1 | 2 | 1.4434 | 0.3333 | 2.3270 | 0.8492 | 6.6250 |
| 30 | 0 | 0 | 0 | 0 | 0 | 0 | 0 | 1.1016 | 0.5000 | 2.6688 | 0.7278 | 5.6749 |
| 31 | 0 | 0 | 0 | 1 | 0 | 0 | 1 | 0.8125 | 0.3333 | 2.9579 | 0.8244 | 8.4154 |
| 32 | 1 | 0 | 0 | 0 | 0 | 1 | 2 | 0.1883 | 0.3333 | 3.5821 | 0.0738 | 4.7708 |
| 33 | 0 | 0 | 0 | 0 | 0 | 0 | 0 | 1.1400 | 0.5000 | 2.6304 | 0.0014 | 4.9376 |
| 34 | 0 | 0 | 0 | 0 | 0 | 0 | 0 | 1.0444 | 0.5000 | 2.7260 | 0.8658 | 7.6525 |
| 35 | 0 | 1 | 0 | 0 | 0 | 0 | 1 | 0.9128 | 0.5000 | 2.8576 | 0.0034 | 7.3876 |
| 36 | 0 | 0 | 0 | 0 | 0 | 0 | 0 | 1.2377 | 0.8333 | 2.5327 | 0.6435 | 2.7233 |
| 37 | 0 | 1 | 0 | 0 | 0 | 0 | 1 | 1.2561 | 0.6667 | 2.5143 | 0.1033 | 2.5109 |
| 38 | 0 | 1 | 0 | 0 | 0 | 0 | 1 | 0.9297 | 0.1667 | 2.8407 | 0.8996 | 12.2256 |
| 39 | 0 | 0 | 0 | 0 | 0 | 0 | 0 | 1.6547 | 0.6667 | 2.1157 | 0.1795 | 1.6164 |
| 40 | 0 | 0 | 0 | 0 | 0 | 1 | 1 | 3.0440 | 0.6667 | 0.7264 | 0.3769 | 3.5664 |
| 41 | 0 | 1 | 0 | 1 | 0 | 0 | 2 | 0.8023 | 0.6667 | 2.9681 | 0.4315 | 12.2880 |
| 42 | 0 | 0 | 0 | 0 | 1 | 0 | 1 | 1.5101 | 0.8333 | 2.2603 | 0.7405 | 3.1274 |
| 43 | 0 | 1 | 0 | 0 | 0 | 0 | 1 | 1.4768 | 0.5000 | 2.2936 | 0.6501 | 7.5649 |
| 44 | 0 | 0 | 0 | 2 | 0 | 0 | 2 | 1.1028 | 0.6667 | 2.6676 | 0.0074 | 6.1294 |
| 45 | 0 | 0 | 1 | 1 | 1 | 0 | 2 | 3.1413 | 0.8333 | 0.6291 | 0.1432 | 3.0044 |
| 46 | 0 | 0 | 0 | 0 | 0 | 0 | 0 | 1.5163 | 0.6667 | 2.2541 | 0.0047 | 2.3248 |
| 47 | 0 | 0 | 1 | 1 | 0 | 0 | 1 | 1.6046 | 0.6667 | 2.1658 | 0.6464 | 3.7816 |
| 48 | 0 | 0 | 0 | 0 | 0 | 0 | 1 | 1.6680 | 0.6667 | 2.1024 | 0.5162 | 4.7001 |
| 49 | 0 | 0 | 0 | 0 | 0 | 0 | 0 | 1.5576 | 0.6667 | 2.2128 | 0.0048 | 5.4577 |
| 50 | 0 | 0 | 0 | 1 | 0 | 0 | 1 | 1.0171 | 0.6667 | 2.7533 | 0.0230 | 2.9148 |
| 51 | 0 | 0 | 0 | 1 | 0 | 0 | 1 | 3.0948 | 0.5000 | 0.6756 | 0.8264 | 1.4857 |
| 52 | 0 | 0 | 0 | 0 | 0 | 0 | 0 | 1.9525 | 0.5000 | 1.8179 | 0.4891 | 2.0343 |
| 53 | 0 | 0 | 2 | 0 | 0 | 0 | 2 | 1.3663 | 0.5000 | 2.4041 | 0.4994 | 4.0725 |
| 54 | 0 | 0 | 0 | 0 | 0 | 0 | 0 | 1.3520 | 0.6667 | 2.4184 | 0.0048 | 3.5979 |
| 55 | 0 | 0 | 0 | 0 | 0 | 0 | 0 | 1.0915 | 0.6667 | 2.6789 | 0.0225 | 3.8804 |
| 56 | 0 | 0 | 0 | 0 | 0 | 0 | 0 | 0.2045 | 0.5000 | 3.5659 | 0.0706 | 8.2023 |
| 57 | 0 | 0 | 2 | 0 | 0 | 0 | 2 | 3.7704 | 0.6667 | 0.0000 | 0.1170 | 2.1574 |
| 58 | 0 | 0 | 1 | 0 | 0 | 0 | 1 | 1.7905 | 0.5000 | 1.9799 | 0.0237 | 2.3448 |
| 59 | 0 | 0 | 0 | 0 | 0 | 0 | 0 | 1.4177 | 0.6667 | 2.3527 | 0.0017 | 1.6603 |
| 60 | 0 | 0 | 0 | 0 | 0 | 0 | 0 | 1.1592 | 0.3333 | 2.6112 | 0.0054 | 1.8922 |
| 61 | 0 | 0 | 0 | 0 | 0 | 0 | 0 | 1.1083 | 0.5000 | 2.6621 | 0.0372 | 1.0153 |
| 62 | 0 | 0 | 1 | 0 | 0 | 0 | 1 | 0.2296 | 0.5000 | 3.5408 | 0.0549 | 5.3269 |
| 63 | 0 | 0 | 0 | 0 | 0 | 0 | 0 | 3.6476 | 0.5000 | 0.1228 | 0.0209 | 2.4977 |
| 64 | 0 | 0 | 0 | 0 | 0 | 0 | 0 | 2.6166 | 0.5000 | 1.1538 | 0.0028 | 3.4578 |
| 65 | 0 | 1 | 0 | 0 | 0 | 0 | 1 | 1.6683 | 0.3333 | 2.1021 | 0.0032 | 3.9353 |
| 66 | 0 | 0 | 0 | 0 | 0 | 0 | 0 | 1.2568 | 0.6667 | 2.5136 | 0.0637 | 5.1657 |
| 67 | 0 | 0 | 0 | 1 | 0 | 0 | 1 | 0.9922 | 0.6667 | 2.7782 | 0.0066 | 2.9079 |
| 68 | 0 | 0 | 0 | 0 | 0 | 0 | 0 | 0.0760 | 0.5000 | 3.6944 | 0.0086 | 2.0769 |
| 69 | 0 | 0 | 1 | 0 | 0 | 0 | 1 | 3.3177 | 0.5000 | 0.4527 | 0.0021 | 1.9730 |
| 70 | 0 | 0 | 0 | 0 | 0 | 0 | 0 | 2.3400 | 0.5000 | 1.4304 | 0.0033 | 1.9270 |
| 71 | 0 | 0 | 0 | 0 | 0 | 0 | 0 | 1.4232 | 0.6667 | 2.3472 | 0.0742 | 4.2114 |
| 72 | 0 | 0 | 0 | 1 | 0 | 0 | 1 | 1.1123 | 0.6667 | 2.6581 | 0.0048 | 2.0872 |
| 73 | 0 | 0 | 0 | 0 | 0 | 0 | 0 | 0.0749 | 0.5000 | 3.6955 | 0.0105 | 2.3877 |
| 74 | 0 | 0 | 0 | 0 | 0 | 0 | 0 | 0.0541 | 0.3333 | 3.7163 | 0.0088 | 3.4705 |
| 75 | 0 | 0 | 0 | 0 | 0 | 0 | 0 | 1.9515 | 0.5000 | 1.8189 | 0.0069 | 1.5660 |
| 76 | 0 | 0 | 0 | 0 | 1 | 0 | 1 | 1.0532 | 0.5000 | 2.7172 | 0.0080 | 1.8711 |
| 77 | 0 | 0 | 0 | 0 | 1 | 0 | 1 | 0.6199 | 0.6667 | 3.1505 | 0.0050 | 5.8200 |
| 78 | 0 | 0 | 0 | 0 | 1 | 0 | 1 | 0.1759 | 0.3333 | 3.5945 | 0.0086 | 3.2581 |
| 79 | 0 | 0 | 0 | 0 | 0 | 0 | 0 | 0.0494 | 0.5000 | 3.7210 | 0.0112 | 2.6312 |
| 80 | 1 | 0 | 0 | 0 | 0 | 0 | 1 | 0.0622 | 0.3333 | 3.7082 | 0.0058 | 5.5635 |

All the variables in the table were defined in detail in the revision.
